# Visual acuity and ocular comorbidities in patients aged 100 years and older: A retrospective, crosssectional study

**DOI:** 10.1007/s00417-025-06947-x

**Published:** 2025-08-28

**Authors:** Takashi Ono, Takuya Iwasaki, Toshihiro Sakisaka, Yosai Mori, Ryohei Nejima, Takashi Miyai, Kazunori Miyata

**Affiliations:** 1 Miyata Eye Hospital, 6-3, Kuraharacho, Miyakonojo, 885-0051 Miyazaki Japan; 2 Department of Ophthalmology, the University of Tokyo, Tokyo, Japan

**Keywords:** Centenarians, Visual acuity, Ocular comorbidities, Cataract, Uveitis, Dry eye

## Abstract

**Purpose:**

The centenarian population is increasing with an increase in global life expectancy. However, data on visual status and ocular diseases in this age group remain limited. We aimed to assess visual acuity and ocular comorbidities in patients aged ≥ 100 years.

**Methods:**

This retrospective cross-sectional study included patients aged ≥ 100 years who visited Miyata Eye Hospital between January 2016 and December 2024. Data on best-corrected visual acuity (BCVA), intraocular pressure, and ocular comorbidities were extracted from medical records. Multivariate linear regression was performed to analyse the associations between BCVA and ocular diseases.

**Results:**

This study included 50 eyes of 25 patients. The mean age was 100.8 ± 1.0 years, and 80.0% of the eyes belonged to women. The mean BCVA was 1.01 ± 0.93 logarithm of the minimum angle of resolution. The most common ocular conditions were dry eye (54%), glaucoma (46%), cataract (40%), and macular degeneration (40%). Multivariate analysis revealed that cataract (*p* = 0.019) and uveitis (*p* = 0.003) were significantly linked to poor visual acuity.

**Conclusion:**

Cataracts and uveitis were the most significant factors contributing to visual impairment in centenarian patients. Thus, an improved understanding of the ocular health status is crucial for maintaining visual function and quality of life in this unique population.

## Key messages

*****What is known?***:**

- The centenarian population is increasing with an increase in global life expectancy; however, knowledge regarding the visual status and ocular diseases in this age group is limited.

*****What is new?***:**

- This retrospective cross-sectional study included patients aged ≥100 years, and data on best-corrected visual acuity, intraocular pressure, and ocular comorbidities were extracted from medical records.
- Cataracts and uveitis were the most significant factors contributing to visual impairment in centenarian patients.
- Thus, an improved understanding of the ocular health status is crucial for maintaining visual function and quality of life in this unique population.

## Introduction

The global population of individuals aged ≥ 100 years—commonly referred to as centenarian patients—is steadily increasing because of advances in healthcare and improved living standards [1]. The number of centenarian patients is expected to reach over 3 million by 2050, according to the United Nations, making this group a crucial demographic in geriatric medicine [2]. Despite this trend, limited data are available regarding the ophthalmic status and visual function of individuals in this age group. Understanding the ocular characteristics of centenarian patients is essential, given that vision plays a crucial role in maintaining the independence and quality of life of older individuals.

Previous studies have examined visual acuity and eye diseases in older individuals, particularly those in their 80 s and 90 s [3–6]. However, few studies have focused on patients aged ≥ 100 years, a group that may present unique clinical challenges due to accumulated ocular and systemic comorbidities, frailty, and limitations in treatment eligibility or willingness [7, 8]. Moreover, the prevalence and impact of age-related ocular conditions, such as cataracts, glaucoma, dry eye disease, and age-related macular degeneration, remain poorly understood in this age group. Although fewer people lived to 100 years or older in the past, recent trends show that there were more than 95,000 centenarians in Japan in 2024, with the number gradually increasing.

In this study, we aimed to examine the visual acuity and ocular comorbidities of patients aged ≥ 100 years who visited a single ophthalmology centre in Japan. We aimed to identify the most prevalent conditions influencing visual function and assess their relative contributions to visual impairment in this understudied population.

## Methods

### Study design and setting

This retrospective, cross-sectional study was conducted at Miyata Eye Hospital in Miyazaki, Japan, a tertiary-care ophthalmology centre, from January 2016 to December 2024. This study adhered to the tenets of the Declaration of Helsinki and was approved by the Institutional Review Board of Miyata Eye Hospital (approval number CS-416-012). Informed consent was acquired through the opt-out method from all patients included in the study.

### Participants

We included patients who were aged ≥ 100 years at the time of their visit to Miyata Eye Hospital between January 2016 and December 2024. This study included only patients with complete ophthalmological examination records. Both eyes meeting the inclusion criteria were included in the analysis. If only one eye had complete records, the patient was excluded from the study.

### Data collection

Demographic and clinical data were extracted from medical records. Collected variables included age; sex; best-corrected visual acuity (BCVA); intraocular pressure; and the presence or absence of cataract, glaucoma, uveitis, optic nerve atrophy, dry eye disease, nasolacrimal duct obstruction, blepharitis, and other retinal disorders at the last hospital visit. BCVA was measured using Landolt C–charts and converted to the logarithm of the minimum angle of resolution (logMAR) for analysis. Intraocular pressure was assessed by noncontact or Goldmann applanation tonometry. Only examination results from the final visit were included.

### Statistical analysis

Descriptive statistics are used to summarise demographic and clinical characteristics. Continuous variables are presented as means ± standard deviations, and categorical variables are presented as counts and percentages. A forced entry multivariate linear regression model was used to identify factors linked to visual acuity, with BCVA (logMAR) as the dependent variable and the presence of individual ocular diseases as independent variables. Statistical significance was set at a p-value of < 0.05. All statistical analyses were performed using GraphPad Prism 9.5.1 (GraphPad Software, San Diego, CA, USA).

## Results

### Patient characteristics

**Fig. 1:**
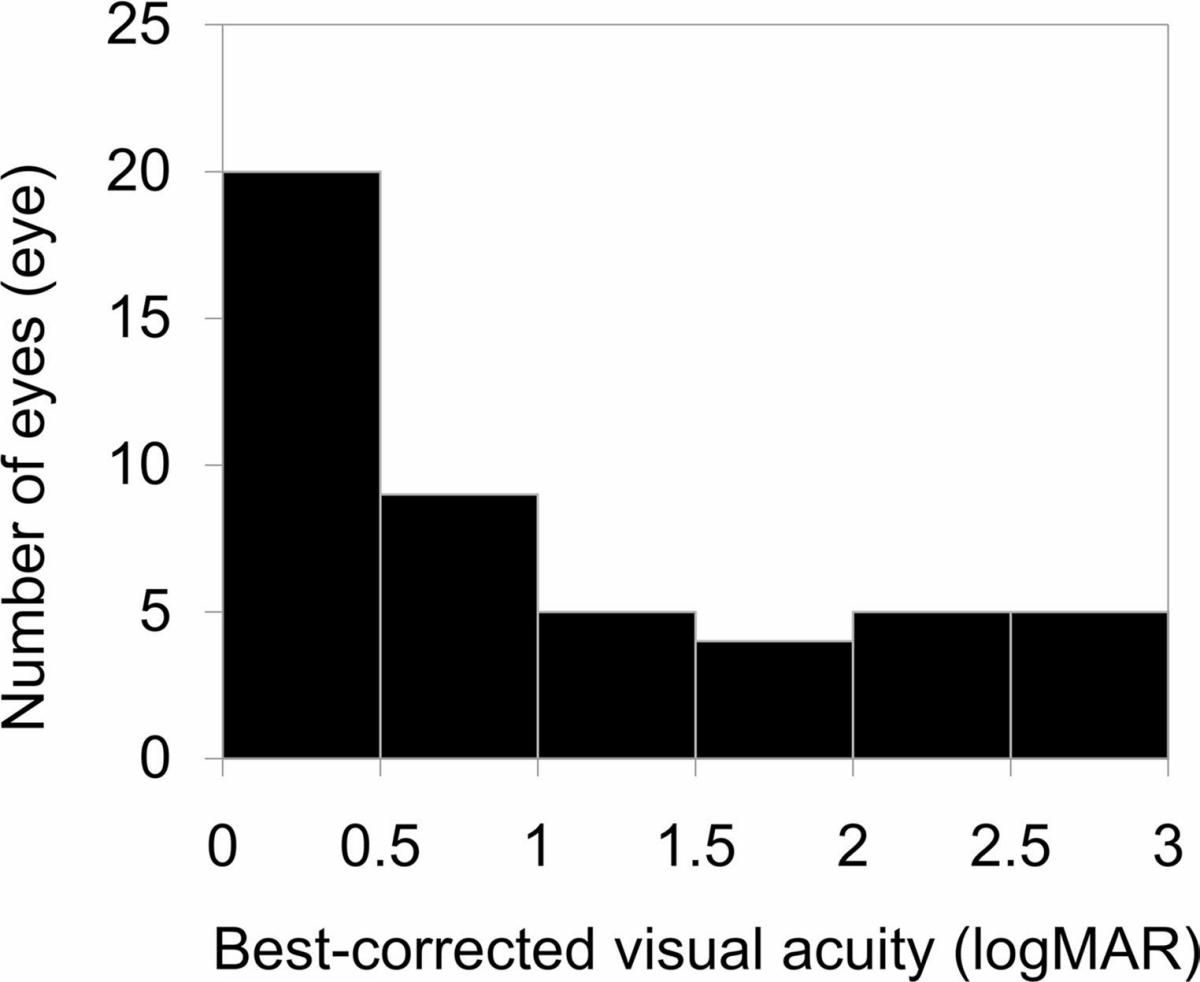
Distribution of best-corrected visual acuity (BCVA) among the centenarian patients The most common BCVA was 0–0.5 logarithm of the minimum angle of resolution (logMAR). The mean BCVA was 1.01 ± 0.93 logMAR

A total of 25 patients (50 eyes) aged ≥ 100 years were included in this study. The mean age was 100.8 ± 1.0 years. Of the 50 eyes analysed, 40 (80%) belonged to women and 10 (20%) to men. The mean intraocular pressure was 13.8 ± 6.7 mmHg, and the mean BCVA was 1.01 ± 0.93 logMAR (approximately 20/200 Snellen). Figure 1 illustrates the BCVA distribution among participants. The most frequent BCVA range was 0–0.5 logMAR (20 eyes; 40%), corresponding to 20/20–20/63 Snellen.

### Ocular comorbidity prevalence

**Fig. 2:**
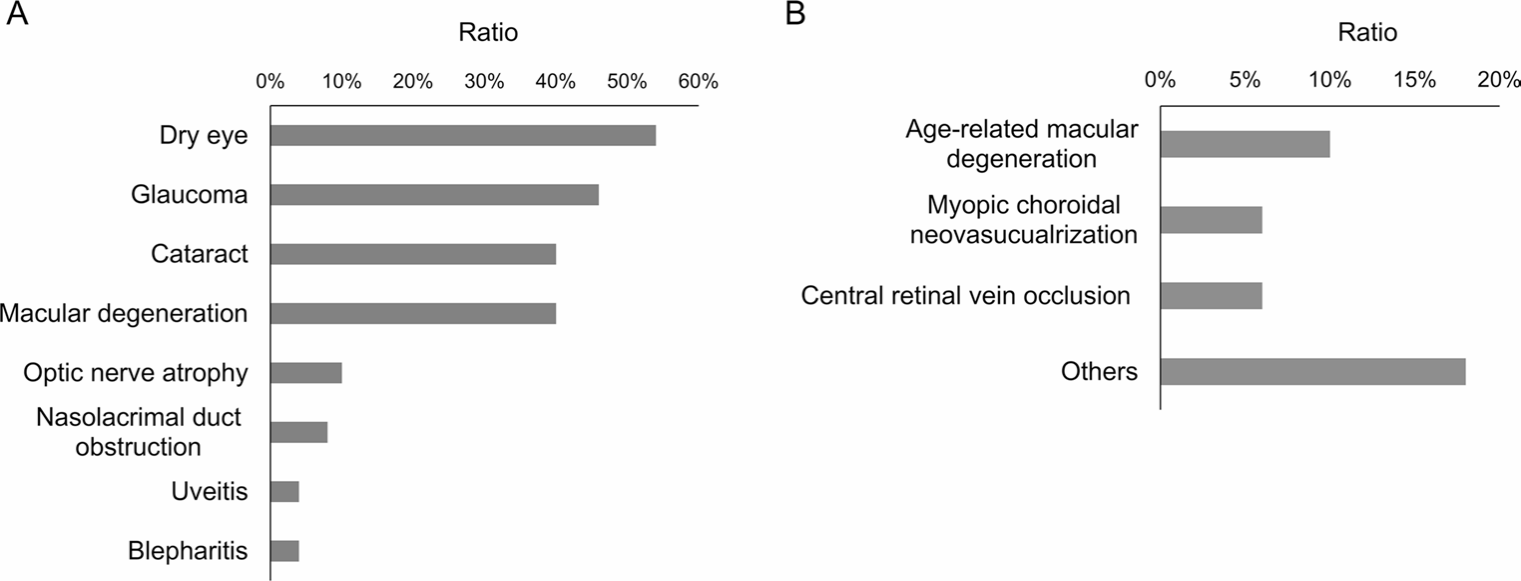
Centenarian patients’ ocular comorbidities **A**. Prevalence of ocular disorders among centenarian patients. Dry eye was the most prevalent condition. Glaucoma, cataracts, and macular degeneration were the next most common ocular diseases observed **B**. Distribution of macular degeneration subtypes among patients. Age-related macular degeneration was the most commonly observed

Several patients had multiple concurrent ocular comorbidities. The most frequently noted ocular conditions were dry eye disease (Fig. 2) (27 eyes; 54%), glaucoma (23 eyes; 46%), cataract (20 eyes; 40%), macular degeneration (20 eyes; 40%), optic nerve atrophy (5 eyes; 10%), nasolacrimal duct obstruction (4 eyes; 8%), uveitis (2 eyes; 4%), and blepharitis (2 eyes; 4%). Glaucoma included angle closure glaucoma (6 eyes; 12%), open angle glaucoma (9 eyes; 18%), normal tension glaucoma (4 eyes; 8%), pseudoexfoliation glaucoma (2 eyes; 4%), and unclassified cases (2 eyes; 4%). Macular degeneration cases included age-related macular degeneration (5 eyes; 10%), myopic choroidal neovascularisation (3 eyes; 6%), central retinal vascular occlusion (2 eyes; 4%), post-surgical epiretinal membrane (1 eye; 2%), cystic macular oedema (1 eye; 2%), and unknown causes (8 eyes; 16%). Both eyes with uveitis presented with non-granulomatous anterior uveitis without vitreous opacity. Blood test results were unremarkable, and we found no evidence of Vogt-Koyanagi-Harada disease, sympathetic ophthalmia, tuberculosis-related uveitis, or infectious uveitis.

All patients who underwent cataract surgery received intraocular lenses insertion, and those who did not undergo cataract surgery still had cataracts. Four eyes (8%) had bullous keratopathy; one eye (2%) was treated with penetrating keratoplasty, and another eye (2%) was treated with Descemet’s stripping automated endothelial keratoplasty.

### Association between comorbidities and visual acuity

The relationship between comorbidities and BCVA in centenarian patients is summarised in Table 1. Among the included eyes, those that underwent cataract surgery with intraocular lens insertion had significantly better BCVA than those who did not (BCVA 0.803 ± 0.819 vs. 1.403 ± 1.002, *p* = 0.013). Dry eye and uveitis were also significantly related to BCVA (*p* = 0.029 and 0.017, respectively).

**Table 1:** Association between comorbidities and best-corrected visual acuity

Condition	Best corrected visual acuity of (-) group	Best corrected visual acuity of (+) group	*p*-value
Dry eye	1.301 ± 0.964	0.787 ± 0.857	0.029*
Glaucoma	0.863 ± 0.803	1.204 ± 1.064	0.423
Cataract	0.803 ± 0.819	1.403 ± 1.002	0.013*
Macular degeneration	0.853 ± 0.883	1.236 ± 0.974	0.142
Optic nerve atrophy	0.964 ± 0.934	1.018 ± 0.942	0.876
Nasolacrimal duct obstruction	0.974 ± 0.863	1.438 ± 1.632	0.890
Uveitis	0.932 ± 0.866	2.850 ± 0.071	0.017*
Blepharitis	1.023 ± 0.950	0.760 ± 0.085	0.825

* *p* < 0.05

Cataract (*p* = 0.019) and uveitis (*p* = 0.003) were both significantly associated with worse BCVA in multivariate linear regression analysis (Table 2). Data on ocular comorbidities are summarised in Table 3. In total, 39 eyes (78%) had more than one ocular condition impairing visual function.

**Table 2:** Multivariate linear regression analysis of the factors associated with best-corrected visual acuity

Condition	Estimate	Standard Error	*p*-value
Cataract (+)	0.5703	0.2347	0.019*
Uveitis (+)	1.8690	0.6012	0.003**

* *p* < 0.05, ** *p* < 0.01

**Table 3:** List of all the patients

Patient No.	Laterality	Age	Sex	Best-corrected visual acuity (logMAR)	Cataract	Intraocular lens	Glaucoma	Optic nerve atrophy	Blepharitis	Dry eye	Nasolacrimal duct obstruction	Uveitis	Macular degeneration	Others
1	L	101	m	0.70	-	+	PEG	+	-	-	-	-	-	
1	R	101	m	0.15	-	+	PEG	+	-	-	-	-	-	
2	L	100	f	0.22	-	+	OAG	-	-	+	-	-	-	
2	R	100	f	0.15	-	+	OAG	-	-	+	-	-	-	
3	L	100	f	1.30	-	+	-	-	-	+	-	-	+	
3	R	100	f	0.15	-	+	-	-	-	+	-	-	-	
4	R	101	f	0.40	+	-	NTG	-	-	+	-	-	-	
4	L	101	f	0.10	+	-	NTG	-	-	+	-	-	-	
5	L	101	f	2.80	-	+	OAG	-	-	+	+	+	+	Myopic choroidal neovascularization
5	R	101	f	2.90	-	+	OAG	-	-	+	+	+	+	Myopic choroidal neovascularization
6	R	103	f	0.40	+	-	Gla	-	-	-	-	-	+	
6	L	103	f	2.90	+	-	Gla	-	-	-	-	-	+	Central retinal vein occlusion
7	L	100	f	0.05	-	+	-	-	-	+	+	-	-	
7	R	100	f	0.00	-	+	-	-	-	+	+	-	+	Choroidal neovascularization
8	R	100	f	1.85	+	-	-	-	-	-	-	-	+	Myopic choroidal neovascularization
8	L	100	f	0.30	-	+	-	-	-	+	-	-	+	
9	L	101	f	1.52	-	+	OAG	+	-	-	-	-	+	Central retinal vein occlusion
9	R	101	f	0.15	-	+	OAG	+	-	-	-	-	-	
10	L	100	f	0.40	-	+	-	-	-	+	-	-	+	
10	R	100	f	0.30	-	+	-	-	-	+	-	-	+	
11	R	104	m	unassessable	+	-	NTG	-	-	-	-	-	-	
11	L	104	m	unassessable	+	-	NTG	-	-	-	-	-	-	
12	L	101	f	2.00	+	-	ACG	-	-	-	-	-	-	Acute glaucoma attack
12	R	101	f	1.00	+	-	ACG	-	-	-	-	-	-	
13	L	101	f	0.70	-	+	-	-	-	+	-	-	+	Post-epiretinal membrane removing surgery
13	R	101	f	0.30	-	+	-	-	-	+	-	-	-	
14	R	101	m	0.52	+	-	-	-	-	+	-	-	+	Age-related macular degeneration
14	L	101	m	0.52	+	-	-	-	-	+	-	-	+	Age-related macular degeneration
15	R	100	f	1.10	-	+	OAG	-	-	-	-	-	+	Age-related macular degeneration
15	L	100	f	1.85	-	+	OAG	-	-	-	-	-	+	Age-related macular degeneration
16	L	102	m	0.70	-	+	-	-	-	+	-	-	-	Post-Descemet’s membrane automated endothelial keratoplasty
16	R	102	m	1.30	-	+	OAG	-	-	+	-	-	-	Post-penetrating keratoplasty
17	R	100	f	0.05	+	-	ACG	-	-	-	-	-	+	
17	L	100	f	2.90	+	-	ACG	-	-	-	-	-	-	Bullous keratopathy, corneal opacity
18	R	100	f	2.00	-	+	-	-	-	+	-	-	+	Cystic macular edema, bullous keratopathy
18	L	100	f	0.10	-	+	-	-	-	+	-	-	-	
19	R	100	f	0.30	-	+	-	-	-	+	-	-	-	
19	L	100	f	0.30	-	+	-	-	-	+	-	-	-	
20	L	102	f	1.00	-	+	-	-	-	+	-	-	+	
20	R	102	f	2.30	+	-	-	+	-	+	-	-	+	
21	R	100	m	0.40	-	+	ACG	-	-	-	-	-	-	
21	L	100	m	2.30	+	-	ACG	-	-	-	-	-	-	Acute glaucoma attack
22	R	100	f	0.82	+	-	-	-	+	-	-	-	-	Meibomian gland dysfunction
22	L	100	f	0.70	+	-	-	-	+	-	-	-	-	Meibomian gland dysfunction
23	L	101	f	2.90	-	-	-	-	-	-	-	-	-	Phthisis
23	R	101	f	2.30	+	-	-	-	-	-	-	-	-	Globe rupture, corneal oedema
24	R	100	f	0.30	-	+	-	-	-	+	-	-	-	
24	L	100	f	1.85	-	+	-	-	-	+	-	-	-	Sebaceous gland carcinoma
25	L	100	f	0.82	+	-	-	-	-	-	-	-	-	Entropion, oculomotor nerve palsy
25	R	100	f	0.52	+	-	-	-	-	-	-	-	-	Entropion

*OAG *open angle glaucoma, *ACG *angle closure glaucoma, *PEG* pseudo-exfoliation glaucoma, *NTG* normal tension glaucoma, *Gla* glaucoma

## Discussion

This study provides a focused assessment of visual function and ocular comorbidities in centenarian patients, a rapidly growing but understudied demographic in ophthalmology. Our findings indicate markedly reduced visual acuity in individuals aged ≥ 100 years, with a mean BCVA of 1.01 ± 0.93 logMAR (approximately 20/200 Snellen). These results align with population-based studies reporting a progressive decline in acuity beyond the ninth decade of life [1, 9–11]. The prevalences of dry eye (54%), glaucoma (46%), cataracts (40%), and macular degeneration (40%) reflect established trends in ocular morbidity within the centenarian population.

A notable finding in our cohort was the strong link between cataract, uveitis, and reduced BCVA. In general, cataracts progress with age, and many patients undergo cataract surgery in middle to older age. Considering age, it is likely that all centenarian patients included in this study had cataracts, but unexpectedly many had not undergone cataract surgery. Clinical notes frequently cited reluctance among patients or families, often driven by concerns regarding surgical risks, limited perceived benefits at an advanced age, and difficulties in postoperative care. This hesitation aligns with previous literature describing barriers to cataract surgery in older populations, especially among nonagenarians [4, 12]. However, emerging evidence supports the safety and efficacy of cataract surgery in centenary individuals because cataract surgery offers considerable gains in quality of life and functional independence [7, 13–15]. These results highlight the requirement for patient-centred counselling that considers chronological age and functional goals, systemic health, and social support structures.

Although less prevalent (4%), uveitis has emerged as a strong predictor of visual impairment. Affected eyes presented with non-granulomatous anterior uveitis and intraocular pressure elevated above 50 mmHg, causing remarkable optic nerve damage. Uveitis can impair vision through multiple mechanisms, including anterior synechiae, vitreous opacities, macular oedema, macular neovascularisation, and macular scarring secondary to choroidal inflammation. The disproportionate impact of inflammatory eye disease in this age group suggests heightened susceptibility to irreversible damage, even after transient inflammatory episodes [16]. The structural fragility of ocular tissues and diminished reparative capacity with age may increase the risk of vision loss in centenarian patients with uveitis. Prompt diagnosis through systematic evaluation and appropriate anti-inflammatory therapy is crucial yet often challenging due to atypical presentations or diagnostic overshadowing by other ocular conditions [17]. However, fluorescein angiography may be difficult to perform in older patients because of systemic comorbidities, complicating the diagnosis of some retinal diseases in this population.

Although glaucoma was highly prevalent in our study population, it showed no significant association with BCVA. This finding may reflect the typical glaucoma-related progression in which peripheral visual field deficits precede central acuity loss [18]. We did not assess visual field results because many patients were unable to complete Humphrey testing, possibly due to limited sustained attention. Moreover, centenarian patients with glaucoma maintained stable disease under long-term topical therapy and declined further surgical intervention. A prior report indicated a glaucoma prevalence of 2.56% among individuals aged ≥ 40 years [19]. The substantially high prevalence in our centenarian cohort underscores the need for routine screening and vigilant monitoring, as undiagnosed or undertreated glaucoma can lead to progressive vision loss [11].

Dry eye disease, although not substantially correlated with BCVA in multivariate analysis, remains a clinically crucial contributor to visual discomfort, fluctuating vision, and reduced quality of life [20, 21]. Our results demonstrated that the prevalence was 54%, higher than a previous study reporting 11.4%, 13.1%, and 14.6% in patients aged 60–69, 70–79, and ≥ 80 years, respectively [22]. In older patients, factors such as decreased blink rate, lid laxity, and systemic inflammatory conditions exacerbate dry eye symptoms [20, 21, 23]. In particular, antihistamines, sedatives, and diuretics used especially by older patients can exacerbate dry eyes. The multifactorial nature of dry eyes in this population requires a tailored multidisciplinary management approach.

Our study highlights the need for geriatric-focused ophthalmic care. Centenarian patients often present with complex medical histories, polypharmacy, cognitive decline, and frailty—all factors that complicate ocular management [24, 25]. Standard interventions may require modification to align with these patients’ priorities and capabilities. For instance, preservative-free eye drops, simplified treatment regimens, and home-based visual rehabilitation can considerably improve adherence and outcomes. Additionally, it is increasingly important to assess the psychosocial impact of visual impairment in this age group. Vision loss in older individuals is strongly associated with depression, social isolation, falls, and institutionalization [26]. Visual rehabilitation and psychosocial support should be integrated into a comprehensive geriatric care framework.

This study has some limitations. The retrospective design and relatively small sample size limit the statistical power and generalisability of the findings. Moreover, a selection bias may have been introduced owing to the data being extracted from a single tertiary-care centre. Further, regarding retinal examination, in some elderly patients, spinal deformities made optical coherence tomography examinations challenging. The absence of standardised grading systems for ocular disease severity and the lack of functional vision measures (e.g., contrast sensitivity, near visual acuity, or visual fields) constrain our ability to evaluate the complete impact of ocular comorbidities. Prospective multicentre studies with larger cohorts and comprehensive functional evaluations are required to validate and expand upon our results.

## Conclusions

Cataract and uveitis are major contributors to vision loss in centenarian patients, while glaucoma and dry eye disease also impair visual function and comfort. As global longevity increases, ophthalmologists must adopt interdisciplinary, individualised, agesensitive care approaches tailored to centenarians. Early detection and management of treatable ocular conditions, combined with holistic support, can remarkably enhance quality of life for this population.

Our findings carry important public health implications. With rising global life expectancy, the centenarian population is projected to grow substantially, particularly in ageing societies such as Japan. Health systems must address the specific needs of oldestold individuals by allocating resources for ageadapted ophthalmic services, ensuring infrastructure for safe surgeries in advanced age, and training ophthalmologists in geriatric care principles.

## Data Availability

The datasets used and/or analysed during the current study are available from the corresponding author upon reasonable request.
